# A Comparative Study on Antiretroviral Therapy (ART) Adherence Among the Tribal and Non-tribal Populations Living With HIV/AIDS in Ranchi, India

**DOI:** 10.7759/cureus.66243

**Published:** 2024-08-05

**Authors:** Anit Kujur, Mithilesh Kumar, Santosh K Soren, Neha Priya, Abhay Kumar, Vidya Sagar, Atul Kachhap, Najam Khalique, Vishwanath Kumar

**Affiliations:** 1 Community Medicine, Rajendra Institute of Medical Sciences, Ranchi, IND; 2 Public Health, Rajendra Institute of Medical Sciences, Ranchi, IND; 3 General Medicine, Rajendra Institute of Medical Sciences, Ranchi, IND; 4 Preventive Medicine, Rajendra Institute of Medical Sciences, Ranchi, IND; 5 Community Medicine, Jawaharlal Nehru Medical College & Hospital, Aligarh Muslim University, Aligarh, IND; 6 Anaesthesiology, Rajendra Institute Of Medical Sciences, Ranchi, IND

**Keywords:** nonadherence, tribals, hiv, aids, adherence, art

## Abstract

Background: Acquired immunodeficiency syndrome (AIDS) is one of the most serious public health exigencies across the globe. However, equally brawny is the global commitment to halt new HIV infections from occurring and ensure that everyone with the disease has access to HIV treatment.

Materials and methods: A cross-sectional analytical study was carried out at an antiretroviral therapy (ART) center in Ranchi, Jharkhand, India, in 2023. Since most patients across the state were registered for ART treatment there, it was selected as the study site. From this center, 30 tribal and 30 non-tribal people were chosen by a random number table. A Microsoft Excel spreadsheet (Microsoft Corp., Redmond, WA) was used to enter the data, and a template was generated. Data were analyzed using IBM SPSS Statistics software for Windows, version 25 (IBM Corp., Armonk, NY). Chi-square and t-test were used to find an association, and a p-value of less than 0.05 was considered statistically significant.

Results: The majority of the patients, i.e., 40%, were from the 40-50 age category with a mean age of 35.25 years, mean weight was 49.41 kg, and mean per capita income was Rs. 2,215.72. We found the prevalence of ART medication adherence among people living with human immunodeficiency virus (PLHIV) to be 88.3%, the degree of nonadherence was 21.7%, and the causes of the nonadherence were long commutes, high travel costs, and wage loss while visiting an ART center.

Conclusion: Despite the best support system by the National AIDS Control Organisation (NACO), which encompasses free drugs, counseling, and reminders, a fifth of patients still were not consistent with their treatments. We need to focus on travel costs and distance to improve this, as these were the main causes of nonadherence to ART.

## Introduction

Acquired immunodeficiency syndrome (AIDS) is one of the most serious public health distresses across the globe. However, equally sturdy is our commitment to prevent new HIV infections from occurring and ensure that all the diseased have access to anti-retroviral treatment. The universal use of antiretroviral therapy (ART) has improved the survival rates of patients, and AIDS has stopped being a fatal disease; it is more like a chronic condition now. In 2021, there were a total of 38.4 million people living with human immunodeficiency virus (PLHIV) and 1.5 million new infection cases all over the world. Of 38.4 million PLHIV, the majority, i.e., 36.7 million, were adults, while 1.7 million were children less than 15 years [1]. According to the Joint United Nations Programme on HIV/AIDS (UNAIDS), globally, 28.7 million people living with HIV were receiving ART, so its coverage was 75%(66%-85%) in 2021 [2].

India has a small proportion (3.3%) of the population living with AIDS [3]. However, it has the second largest population load on the planet with diverse social demographics. If we look proportionally, the number may be insignificant in our country but appears to be alarming when seeing the total number of patients, which amounts to about 2.5 million PLHIV [2]. The introduction of ART has improved the life span of PLHIV, so much that the disease condition has stopped being fatal and is more like a chronic disease [3].

Overcoming the AIDS epidemic has been doable to a substantial extent due to innovations in the diagnosis, prevention, and treatment of the disease. Despite advances in the scientific understanding of HIV, its prevention, and treatment, as well as years of significant effort by the global health community and leading governments, too many people with HIV even now cannot access and afford the prevention and treatment. The AIDS epidemic not only affects individual lives but also affects families, communities, and countries.

The introduction of different levels of ART therapy has changed the course of the disease with well-documented benefits [4,5]. Now ART is started for all infected people, disregarding the CD4 cell count level, so that viral load is suppressed and the development of AIDS is delayed, prolonging survival and reducing the risk of transmission to others [6,7]. Failure to maintain adherence to ART may lead to dangerous outcomes like drug abuse, deterioration of mental health, and communicable diseases like hepatitis. Several studies have shown that the success of ART depends on the extent to which a patient sticks to their treatment, follows the prescribed doses, comes for follow-up on time, and other instructions [8,9]. It has been documented that for ART to have an optimal response, an adherence rate of more than 95% has been accepted [5,10]. Also, non-compliance with ART has been observed to lead to the development of HIV drug resistance [5,10]. Adherence to ART becomes very important in our country because of the absolute number of HIV/AIDS cases, the social and economic levels of patients, their education status, and the regional differences. For example, the majority of HIV/AIDS patients come from low educational backgrounds and do not recognize the importance of continuing ART. They tend to become irregular or stop treatment altogether when their health status improves [11]. This results in interruption of ART; patients restart treatment when adequate funding becomes available [12]. 

This causes a loss of zeal and motivation in patients to maintain optimal levels of health status [13-15]. In this sense, people in smaller communities experience more problems than those in larger cities. As a result, it is difficult for PLHIV to access healthcare services freely. These patients typically choose to self-medicate and do not undergo medical care services because they are worried about the implications of disclosing their illness [16].

Patients’ adherence to antiretroviral medications is a primary determinant of the effectiveness of treatment. It also predicts the clinical course of HIV infection and the development of AIDS. Jharkhand has an adult HIV prevalence ranging from 0.06 to 0.11 and a 0.04 incidence rate per 1,000 normal population [17]. The last study done on ART adherence reported an adherence rate of 57% about 20 years ago [18]. Jharkhand has about 26% tribal population, with 32 tribal groups like Santhal, Munda, and Ho and eight particularly vulnerable tribal groups (PVTGs) like Asur, Birhor, and Mal Pahariyas. This study is intended to compare the relative importance of patient and treatment characteristics on nonadherence behavior. Moreover, there hardly has been much research on these unprivileged classes. So, we performed a comparative study to provide conclusive data on ART adherence across tribal and non-tribal populations in Jharkhand and the reasons for nonadherence (if any) in HIV/AIDS patients in Jharkhand.

## Materials and methods

A cross-sectional analytical study was conducted at the ART center of Rajendra Institute of Medical Sciences, Ranchi, Jharkhand, India. Since it had the largest number of patients from throughout the state registered for ART, it was called our study site. Randomly from this center, a total of 60 patients were included. Out of 60, 30 tribal and 30 non-tribal patients taking treatment for at least six months were selected randomly by a random number table. The study was conducted after the approval of the Institutional Ethics Committee of Rajendra Institute of Medical Sciences with approval number 65 dated May 21, 2022.

The study population was all people above 18 years of age registered at the ART center who had taken a minimum of six months of treatment and were willing to participate after taking proper written informed consent. The study was conducted from November 2022 to April 2023. At each ART center, data of all the patients fulfilling the above criteria were analyzed to get treatment adherence. Adherence was measured by self-reported medication adherence and pharmacy refill data (Appendix A). We tried to look into the reasons behind nonadherence using in-depth interviews after determining the prevalence of adherence and nonadherence. A semi-structured, open-ended questionnaire was used for in-person interviews with each patient, and a comparison of the two groups was made. Thus, a total of 60 patients along with the counselor were interviewed.

A few operational definitions used in our study are as follows: 1) Adherent: all patients consuming ≥95% of the drug prescribed in four weeks (in the last six months) were considered adherent to treatment [19]; 2) Nonadherent: all patients who failed to consume at least 95% of the drug prescribed in four weeks (in the last six months) due to any cause were considered nonadherent to treatment [19]; 3) Tribal: a person was considered tribal if he had a Certificate of Scheduled Tribe to be issued by the Government of Jharkhand or any other documents issued by an authorized person where his ethnicity has been mentioned, or in the absence of a certificate, he will be verified by us according to his cultural practices; 4) Non-tribal: those not fulfilling the criteria for tribal were considered non-tribal.

Data entry and analysis

The generated data were captured into a Microsoft Excel spreadsheet (Microsoft Corp., Redmond, WA), and a template was created and entered into IBM SPSS Statistics software for Windows, version 25 (IBM Corp., Armonk, NY). Simple frequency tables of all the variables were made.

For continuous data, the mean and standard deviation were computed. For both nominal and ordinal data, the proportion was utilized. A substantial statistical association was found using the unpaired t-test and univariate analysis. The independent predictors of ART nonadherence were assessed using binary logistic regression. The level of significance was considered at alpha less than 0.05. A p-value of less than 0.05 was taken as statistically significant.

## Results

In this research, we sought to determine the prevalence of ART medication adherence among PLHIV, the extent of non-compliance, and its associated causes. Table 1 displays the PLHIV's level of ART adherence. It was around 78% among the selected sample of patients. Of the 60 patients, 13 (21.7%) were not taking ART medication. Table 2 shows mean values among the patients who were adherent and nonadherent to ART. The age, weight, blood urea level, serum glutamic-oxaloacetic transaminase (SGOT), hemoglobin, and CD4 count did not show any significant relation between the two groups. The mean serum creatinine level was found to be lower among the nonadherent patients, and this was statistically significant with a p-value of 0.005.

**Table 1:** Distribution of people living with human immunodeficiency virus (PLHIV) according to the antiretroviral therapy (ART) drug adherence

ART drug adherence	Frequency	Percentage
Yes	47	78.3%
No	13	21.7%
Total	60	100.0%

**Table 2:** Mean values among patients who were adherent and nonadherent to antiretroviral therapy *Significant The mean creatinine level was found to be lower among nonadherent patients, and this was found to be statistically significant with a p-value of 0.005.

Variables	Adherent	Nonadherent	F	p-value	95% Confidence interval of the difference	
					Lower	Upper
Age (years)	34.85±11.235	36.92±9.242	0.386	0.545	-8.879	4.735
Weight (kg)	50.43±9.042	48.54±16.435	0.148	0.459	-7.945	3.636
Blood urea level (mmol/L)	21.00±8.485	11.50±1.732	5.842	0.414	-14.219	18.219
Serum creatinine (mg/dL)	1.00±0.212	0.800±0.173	22.857	0.005*	-0.0151	0.415
Serum glutamic-oxaloacetic transaminase (SGOT, U/L)	29.75±6.946	25.50±2.380	1.957	0.291	-4.734	13.234
Hemoglobin (g/dL)	13.25±2.630	19.00±2.630	0.497	1.750	-2.103	5.603
CD4 count (cell/mm3)	403.05±235.825	282.40±161.153	0.900	0.158	-49.661	290.961

Table 3 shows the distribution of adherent and nonadherent patients according to their ethnicity. Table 4 shows missed doses of ART in a month among patients according to ethnicity. The majority (17, 56.7%) were irregular in taking ART medication as they missed their drug dose thrice in a month and this factor was found to be statistically significant with a p-value of 0.036.

**Table 3:** Distribution of adherent and nonadherent patients according to their ethnicity Fisher's exact test = 0.532; p-value = 0.266; odds ratio = 0.550 (0.157-1.931)

Variable		Adherent to antiretroviral therapy		Total; n(%)
		Yes; n(%)	No; n(%)	
Ethnicity	Tribal	22(73.3%)	8(26.7%)	30(100%)
	Non-tribal	25(83.3%)	5(16.7%)	30(100%)
Total		47(78.3%)	13(21.7%)	60(100%)

**Table 4:** The number of missed doses of antiretroviral therapy (ART) in a month divided as per the patient's ethnicity Chi-square value = 30.122; p-value = 0.036*

Variable		Doses of ART missed in a month					Total; n(%)
		Once; n(%)	Twice; n(%)	Thrice; n(%)	Four times; n(%)	None; n(%)	
Ethnicity	Tribal	0(0%)	8(26.7%)	17(56.7%)	2(6.7%)	3(10%)	30(100%)
	Non-tribal	5(16.7%)	11(36.7%)	13(43.3%)	1(3.3%)	0(0%)	30(100%)
Total		5(8.3%)	19(31.7%)	30(50%)	3(5%)	3(5%)	60(100%)

According to ethnicity, the reasons why certain people do not adhere to ART are listed in Table 5. Distance was a barrier that hindered three (37.5%) of the tribe members from receiving ART.

**Table 5:** Distribution of the participants based on the reason for nonadherence to antiretroviral therapy (n = 13)

Reasons for nonadherence	Frequency of tribal participants(%)	Frequency of non-tribal participants (%)
Long-distance travel	3(37.5%)	1(20%)
Long-distance travel and travel expenses	1(12.5%)	0(0.0%)
Too busy doing other things	0(0.0%)	1(20%)
Not getting medicine soon	0(0.0%)	1(20%)
No issue	2(25%)	2(40.0%)
Police custody	1(12.5%)	0(0.0%)
Due to illness	1(12.5%)	0(0.0%)
Total	8(100%)	5(100%)

## Discussion

The human immunodeficiency virus has no effective treatment that can cure the disease. However, it can be managed with proper medical care. We have a good network of the National AIDS Control Organization (NACO), which helps in managing HIV patients as soon as they are diagnosed. Over the past 20 years, the prognosis of HIV patients has improved over the last two decades due to the widespread availability of highly active antiretroviral therapy (HAART). In the past 10 years, the HIV situation has stabilized. In India, the percentage of people living with HIV who receive ART was reported to be 65% in 2021, as per the World Bank collection of development indicators, gathered from official sources [20].

Our current findings indicated high levels of adherence among major chunks of people living with HIV who were receiving ART at tertiary care institutions even working with low resources. Our findings demonstrate that over 75% of people adhered to the medication (Table 1), which was found similar to other developing countries like Senegal (78%) [21] and South Africa (88%) [22,23]. In India, a similar finding was found in the meta-analysis done by Mhaskar et al. [24], in which aggregated results demonstrated an ART adherence rate of 70% (95% confidence interval: 59-81%, I(2) = 96.3%). Analoging this, a study by Naik et al. [15] depicted a 57% adherence rate and concluded that financial constraint was the biggest obstacle.

The findings showed that the mean age among adherent patients was 34.85±11.235 years and among nonadherent patients was 36.92±9.242 years with a mean weight of 50.43±9.042 kg among adherent and 48.54±16.435 kg among nonadherent patients (Table 2). The CD4 count mean was more than 400 cells/mm^3^ among adherent patients as compared to nonadherent patients, where the viral load was less than 300 cells/mm^3^. Similar findings were found in a study done by Morowatisharifabad et al. [25], where nonadherent PLHIVs showed an increased viral load and low CD+ T cell counts. The mean hemoglobin was higher among adherent patients as compared to nonadherent ones. Other biochemical parameters like blood urea and SGOT were also higher when compared to patients who adhered to the treatment. Mean serum creatinine was less than one among adherent patients compared to nonadherent ones. It was statistically significant with a p-value of 0.005 and a 95% confidence interval of -0.0151 to 0.415.

Table 3 demonstrates the distribution of adherent and nonadherent patients according to their ethnicity. Although the tribal patients had a higher nonadherence rate (26.7%) than the non-tribal patients, the difference was not statistically significant. Apart from our study, no such study was found comparing adherence based on ethnicity in Jharkhand.

Table 4 shows the dose of ART medication missed in a month among patients according to the ethnicity of the patient. Over the longer recall period, there was a slight decrease in medication adherence. Among non-tribal patients, over 40% missed their dose three times in a month, while over half of tribal patients missed their dose three times. This difference was found to be statistically significant, with a p-value of 0.036. The main reason for missing the dose was forgetfulness; similar reasons were found in a study done by Morowatisharifabad et al. [25].

Table 5 lists the reasons why certain people do not adhere to ART based on their ethnicity. Patient-related factors accounted for the majority of the reasons for nonadherence to ART. The distance was a barrier that hindered three (37.5%) of the tribal patients from receiving ART. Similar findings were found in the study done by Adeniyi et al. [26] and Morowatisharifabad et al. [25], where one of the most reported reasons for nonadherence was travel outside the place of residence for treatment.

Limitations

Due to budget constraints, our study was done only at one center; it can be expanded to other hard-to-reach tribal areas, also bringing to light their unique problems in maintaining ART adherence. Also, we started with nonadherent patients, of which many turned out to be taking drugs from other centers or privately, further reducing our sample.

## Conclusions

Overall adherence to ART treatment was high compared to other studies; considering ethnicity, it was more common among non-tribals than tribal people. We conclude that long commutes, high travel costs, and financial constraints while visiting an ART center were the main causes of nonadherence to ART. However, the results may not give a complete picture as the study was done with a small sample. Planning a similar survey of nonadherent patients from diverse areas with difficult terrain and the involvement of non-governmental organizations (NGOs) can give a better overview.

## Appendices

Appendix A

Questionnaires Used In the Study 

**Table 6:** Questionnaire to gather sociodemographic data

Patient no.:		Date:	
A. Sociodemographic profile			
S.no.	Questions	Coding categories	Response
A1	Name	Reg. no	
A2	Mobile no.		
A3	Gender	Male (1) Female (2)	
A4	Age (completed years)		
A5	Weight (in kg)		
A6	Religion	Hindu (1) Muslim (2) Christian (3) Sarna (4) Others (specify) (5)	
A7	Ethnicity	Tribal (1) Non-tribal (2)	
A8	Residence	Urban (1) Rural (2)	
A9	Category	Genera l(1) OBC (2) SC (3) ST (4) Others (specify) (5)	

**Table 7:** Questionnaire to gather sociodemographic data part 2

A10	Education	Illiterate (1) Literate (2) Primary (3) Secondary (4) Higher Secondary (5) Graduate & Above (6)						
A11	Occupation	Govt. service (1) Private job (2) Farming (3) Self-employed (4) DailyWages (5) Industrial/Factory worker (6) Student (7) Unemployed (8) Driver (9) Others (specify) (10)						
A12	Marital status	Married (1) Unmarried (2) Divorced (3) Widow/Widower (4)						
A13	Type of family	Nuclear (1) Joint (2)						
A14	Total number of family members	Sex						
		Age						
A15	Total family income (monthly)							
A16	Per capita income (monthly)							
A17	Socio-economic status (A/c to modified BG Prasad’s classification)	Class I, Class II, Class III, ClassIV, ClassV						

**Table 8:** Personal information questionnaire

B. Personal information				
B1	Are you addicted to any drug?	yes (1) No( 2)		
B2	Present addiction to any drug?	yes (1) No (2)		
B3	Past addiction to any drug?	yes (1) No (2)		
B4	If past/present addictions existed, then	how long … (1) Regular (2) Occasional (3)		
B5	Type of addiction	Specify……..		
B6	Do you take alcohol?	Yes (1) No (2)		If no, then refer to Q No C1
B7	If yes, then from when?	Present alcoholic (1) Past alcoholic (2)		
B8	If present, then how frequently?	Daily (1) Once a week (2) Twice a week (3) More than twice a week (4) Once a month (5) Occasional (6) Others (7)		
B9	If present/past, then what amount of alcohol was taken?	0-30ml (1) 30-60ml (2) >60 ml (3)		
B10	Which type of alcohol do you take?	Indigenous (1) Country made (2) Foreign (3) Multiple (4)		
C.Medical history				
C1	Are you also suffering from TB?	Yes(1) No (2)		

C2(a)	Type of TB as per record	Pulmonary TB(+) (1) Pulmonary TB (-) (2) Extra pulmonary TB(3)		
C2(b)	Category of TB as per record?	Cat.I (1) Cat.II( 2) MDR-TB (3)		
C3	Where you were diagnosed as HIV-positive?	Govt. (1) Private (2) Others-----		
C4	When you were diagnosed as HIV-positive?	<1 Years (1) 1-5 Years (2) 5-10 years (3) >10 years (4)		
C5	Are you taking ATT?	Yes (1) No (2)		If no, then specify
C6	What was the mode of transmission?	Sexua l(1) Blood transfusion (2) Needle prick (3) MTCT (4) IV drug abuse (5) Others (6)		
Laboratory investigations				
C7(a)	Current CD4 cell count as per record	<500/cumm (1) >500/cumm (2)		
C7(b)	CD4 testing done as per schedule?	Yes (1) No (2)		
C7(c)	Other baseline investigation done as per record?	Yes (1) No (2)		
C7(d)	Hb %			
	RBC count			
	WBC count			
	RBS			
	SGPT			
	SGOT			
	HBsAg			
	Anti-HCV			
	Serum creatinine			
	Blood urea level			
	WHO clinical stage			
	Functional WAB			
	CD4 count			

**Table 9:** ART drug history

D. ART treatment history			
D1	Are you taking ART?	Yes (1) No (2)	If no, then go to D2 (b)
D2(a)	If yes, then for how long?		
D2(b)	If not, then for how long?		
D3	Specify the reason for not taking ART		
D4	Regimen of ART as per record	First-line ART (1) Second-line ART (2) Third-line ART (3)	
D5	Do you come to take ART as advised schedule?	Yes(1) No(2)	

D6	Any side effects on taking ART?	Yes (1) No (2)	
D7	If yes, then specify		
D8	Any history of STD?	Yes (Specify) (1) No (2)	
D9	Treatment taken?	Yes (1) No (2)	
D10	Any other opportunistic infection present?	Specify	
D11	Treatment taken for it?	Yes (1) No (2)	
D12	Are you suffering from any chronic illness?	Diabetes (1) Hypertension (2) Cardiac disease (3) Any lung disease (4) Any renal disease (5) Others (6) (Specify) No disease associated (7)	
E. Family history			
E1	Any family history of HIV?	Yes (1) No (2)	If no, then ask Q.F
E2	Treatment history of that member?	ART Taken(1) ART NotTaken(2)	
E3	Treatment taken as per advised?	Yes(1) No(2)	
E4	Where was treatment taken?	Govt. setup (1) Pvt. hospital (2) Pvt. clinician (3) NGOs (4) Others (5)	
F. Assessment of adherence to ART			
F1	What is the schedule of distribution of medicines here?	Daily (1) Weekly (2) Monthly (3) Others (4)	
F2	Is the timing of the ART centre compatible with you?	Yes (1) No (2)	
F3	How is the behavior of staff giving medicines at the ART centre?	Good (1) Average (2) Bad (3)	

F4	How long it takes to collect medicines at the ART Centre?	Hours (1) Minutes (2) Others (Specify)		
F5	Do you believe that AIDS is curable?	Yes (1) No (2)		
F6	How frequently do you come here to take medicines?	Regularly (1) Irregularly (2)		
F7	Are you taking the ART medicines as advised?	Yes (1) No (2) If no, then why? (Specify)	If no, then go to G1	
F8	Would you refer other AIDS patients to the ART Centre?	Yes (1) No (2) If no, then why? (Specify)		
G. Causes of Nonadherence				
G1	How many times or doses of ART do you miss in a week?	Once-1 Twice-2 More than Twice-3 Others-4 Specify		
G2	How many times or doses of ART do you miss in a month?	Once-1 Twice-2 More than Twice-3 Others-4 Specify		
G3	What is the cause of irregularity? (Multiple responses possible)	Ignorance/Forgetting(1) Side effects of drugs (2) Felt better and left (3) Taking medicine from another centre (4) Long travellng distance to the DOTS Centre (5) Travel expenses (6) Problem getting leave to come to the DOTS Centre (7) Shortage of drugs (8) Inadequate staff (9) Poor information and communication (10) Social stigma (11) Traditional healers (12) Drug interaction with ART (13) Travel to some other place for work/migrated (14) Social activities (15) Treatment fatigue/Long treatment (16) Long treatment (17) Alcohol consumption (18) Fasting (19) Due to illness (20) Too busy in doing other things (21) Psychiatric problem (22) Others (specify) (23)		
